# Assessing the Reliability and Validity of Inertial Measurement Units to Measure Three-Dimensional Spine and Hip Kinematics During Clinical Movement Tasks

**DOI:** 10.3390/s24206580

**Published:** 2024-10-12

**Authors:** Anna H. Bailes, Marit Johnson, Rachel Roos, William Clark, Harold Cook, Gina McKernan, Gwendolyn A. Sowa, Rakié Cham, Kevin M. Bell

**Affiliations:** 1 Department of Bioengineering, University of Pittsburgh, Pittsburgh, PA 15213, USAkmb7@pitt.edu (K.M.B.); 2 Department of Physical Therapy, University of Pittsburgh, Pittsburgh, PA 15213, USA; 3 Department of Orthopaedic Surgery, University of Pittsburgh, Pittsburgh, PA 15213, USA; 4 Department of Mechanical Engineering, University of Pittsburgh, Pittsburgh, PA 15213, USA; 5 Department of Physical Medicine and Rehabilitation, University of Pittsburgh, Pittsburgh, PA 15213, USA

**Keywords:** inertial measurement units, spine, validity, reliability, low back

## Abstract

Inertial measurement units (IMUs) provide benefits over the traditional optoelectronic motion capture (OMC) systems in measuring kinematics for the low back pain population. The reliability and validity of IMUs to quantify three-dimensional motion for multiple hip/spine segments have not been systematically evaluated. The purpose of this study was to determine the repeated-measures reliability and concurrent validity of an IMU system for measuring the three-dimensional spine/hip kinematics in six common movement assessments. Seventeen participants (32.3 (14.7) years; 11 female) performed two trials each of six range-of-motion assessments while fitted with four IMUs (T1/T2, T12/L1, L5/S1, and femur). The IMUs showed good–excellent reliability for most of the movements in the primary plane and poor–moderate reliability in the non-primary planes. The IMU and OMC systems showed generally good–excellent agreement in the primary plane and RMSE values between 3.03° and 15.75°. The removal of outliers based on the Bland–Altman analysis resulted in RMSE values between 2.44° and 10.30°. The system agreement in the non-primary planes was generally poor–moderate, and the RMSE values ranged from 2.19° to 45.88°. Anomalies in the proprietary sensor fusion algorithm or calibration may have contributed to the large RMSE values, highlighting the importance of assessing data for physiological relevance. The results suggest that these IMUs may be best suited for population-based studies measuring movement in the primary plane and point toward the need for the development of more robust approaches for broader implementation.

## 1. Introduction

In low back pain (LBP), it is important to measure spine movement to evaluate symptom provocation and inform treatment decisions [1,2,3,4,5,6]. Individuals with LBP have altered motion in their lumbar spine [2,6], thoracic spine [2], and lower limbs [7,8,9]. The impaired range-of-motion makes it difficult to complete functional activities and can perpetuate pain and disability in the LBP population [4,5]. It is important to understand the clinical implications of these kinematic changes, but this is difficult given that most motion analysis is representative of only a single time point in an artificial laboratory environment [10].

Optoelectronic motion capture (OMC) is the current gold standard for quantifying spine kinematics [2,11,12]. OMC involves the use of reflective markers—which must be visible to a set of cameras—to track body movement. Unfortunately, reflective markers are easily occluded by clothing or skin [13,14], especially during end-range movements. In addition, OMC is expensive, making it cost-prohibitive in many clinical settings where treatment decisions occur. Finally, OMC is not conducive to ‘real-world’ settings, such as remote monitoring, and requires the use of a specialized laboratory space. Therefore, OMC cannot be used to monitor movement in a clinical or at-home setting. Due to the limitations of the OMC system, small wearable inertial measurement units (IMUs) are gaining popularity for measuring joint kinematics [12,15], including for use in the spine [11,12,16,17,18,19]. Hodges and Hoorn emphasized the need for the remote monitoring of spine kinematics as a vital addition to a proposed ‘suite of sensors’ useful for the future of spine care [10]. Specifically, IMUs are noted as necessary tools in building prediction algorithms for precision medicine applications [20]. IMUs provide additional benefits over the gold-standard OMC systems in that they enable motion tracking over longer time periods and within at-home or community settings [12,15]. The characteristics of LBP (e.g., the propensity for acute flareups [21], symptom fluctuations [22], and prevalence of movement impairments across functional tasks [2,4,23]) make IMUs particularly compelling for monitoring movement across time and tasks in this population.

Before incorporating IMUs into research and clinical settings, it is important to assess their reliability and validity as compared to the gold-standard OMC system [15]. A rigorous kinematics assessment in LBP should include the following components: (1) an analysis of multiple body segments to capture any compensatory movement or maladaptive movement strategies associated with pain [7,9,19] and (2) an assessment of the three-dimensional range of motion to monitor coupled movements (e.g., twisting while lifting) that may perpetuate pain and increase the risk for further injury [24,25]. A systematic review by Papi and colleagues highlighted 22 different studies investigating the use of wearable sensors to study spine kinematics. However, only five [16,17,26,27,28] of the included studies included three-dimensional range of motion as an outcome measure. Of these five studies, only three [16,26,27] captured the three-dimensional motion of multiple body segments (i.e., thoracic, lumbar, and hip). The three studies that examined three-dimensional multi-segment movement focused only on specific dynamic movements (i.e., stair climbing [26,27] or sit-to-stand [16]). To the authors’ best knowledge, no existing studies have rigorously examined three-dimensional multi-segment kinematics across a variety of clinical movements. The current study builds upon the existing literature that explores the use of wearable sensors for measuring spine kinematics [11,12,16,17,18,19]. This study is novel because it rigorously quantifies the repeated-measures reliability and concurrent validity of an inertial measurement unit system to measure three-dimensional kinematics across multiple spine/hip segments for six common clinical movement assessments. Multi-planar and multi-segment analyses are crucial to fully elucidate the biomechanical patterns and injury risk for LBP.

## 2. Materials and Methods

### 2.1. Participants

Participants were recruited after obtaining Institutional Review Board (IRB) approval through the University of Pittsburgh (STUDY20020091). Participants were recruited through the University of Pittsburgh’s Clinical and Translational Science Institute research registry and community flyers. Participants were included if they were between 18 and 70 years old, reported no low back pain in the last two years, and denied exercise or activity restrictions preventing them from completing low back range of motion tests. Participants were excluded if they reported a history of cancer, spinal cord compression, or discitis.

### 2.2. Instrumentation

**Figure 1:**
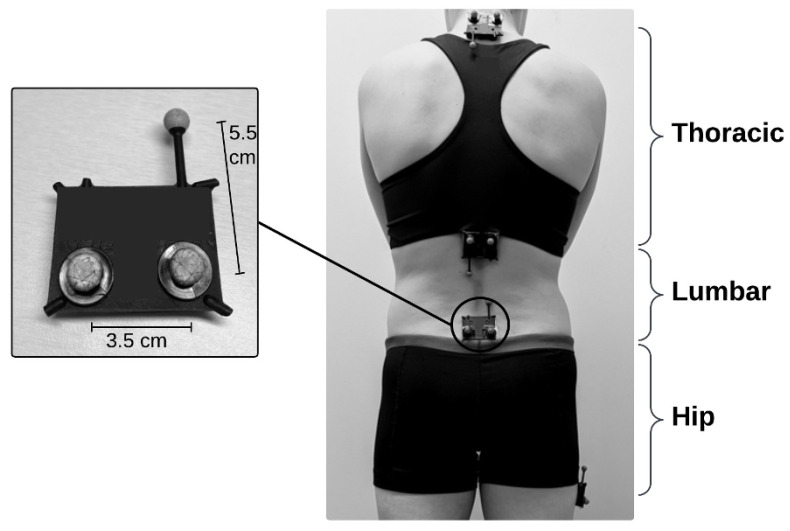
Experimental setup shows IMUs on T1/T2, T12/L1, and L5/S1 interspinous processes and right lateral thigh and corresponding segments. Each IMU has three reflective OMC markers acting as a rigid body cluster attached to a custom black plastic casing. cm = centimeters.

Placement of the four sensors enabled assessment of thoracic, lumbar, and hip motion (Figure 1). Four IMUs (Lifeware Labs, LLC, Pittsburgh, PA, USA) were adhered to each participant using double-sided adhesive body-safe tape (Figure 1). Each IMU sensor was fitted with three reflective markers to capture IMU and OMC data simultaneously. Spine sensors were turned on, three-dimensionally calibrated using a figure-eight waving pattern, and placed at palpated T1/T2, T12/L1, and L5/S1 interspinous spaces. Spine sensors were placed according to the literature, on landmarks that enabled distinction of thoracic and lumbar segments [17,29,30,31,32]. The hip sensor was calibrated in the same fashion and placed on the right femur, in a location chosen to minimize clothing artifact. The hip sensor was located either 10 cm distal to the right greater trochanter or 10 cm proximal to the right lateral femoral epicondyle (depending on participant’s clothing). Variable location of the hip sensor did not create additional variability in the results for two reasons: (1) the OMC markers were adhered directly to the IMU sensors and measured the same movement regardless of location, and (2) peak hip range of motion is not impacted by sensor placement along the femur, which is considered a rigid body for kinematic analysis [33].

The IMU devices included a MEMS IMU (Bosch Sensortec model BNO055) and a Bluetooth transmitter. The IMUs recorded triaxial linear accelerations, triaxial angular velocities, and magnetic field readings. The lab space used for data collection was determined to be free of magnetic interference. Absolute orientation of the sensor was described by quaternions derived using a built-in sensor fusion algorithm, which took place on the IMU (Bosch Sensortec model BNO055). Raw data were wirelessly transmitted to an Android^TM^ smartphone (Pixel 3, Google, LLC, Mountain View, CA, USA) at a rate of 62.5 Hz, and a custom software application (Lifeware Labs, LLC, Pittsburgh, PA, USA) was used for data capture. OMC data were collected at 120 Hz using a Vicon Motion System with 14 T40S cameras (Oxford, UK). After sensors were adhered to the participant, the participant stomped on the floor with their right foot one time. The stomp resulted in a distinct peak in the linear acceleration readings captured by each sensor, which were then used to manually time-synchronize the four IMUs.

### 2.3. Movement Protocol

Participants completed two trials each of six range-of-motion (ROM) tasks to assess the repeated-measures reliability of the IMUs. A trial consisted of five repetitions of six ROM movements: flexion/extension (F/E), left/right axial rotation (L/R AR), and left/right side bend (L/R SB). Participants were instructed to move as far as they could comfortably go while keeping their knees and pelvis straight for each repetition. Participants practiced the movement with feedback from the primary author (A.H.B.) until it was performed per instructions, at which point data collection began for the five repetitions. The extension (E) movement was modified to be only a portion of the participants’ total available range-of-motion as most participants’ full extension range resulted in blocking of the OMC reflective markers. Participants were cued to stop the extension movement once the OMC markers were occluded. The movement tasks were performed in the same order for each participant. Movement trials were also video recorded using a camcorder that was time-synchronized with the OMC system. After completing the first trial (Time 1), the four IMUs were removed from the participant before a short rest period lasting no more than 10 min. Sensors were then reapplied to the previously described landmarks and the movement tests were performed again for a second trial (Time 2). Participants were again instructed to move as far as they could comfortably go while keeping their knees and pelvis straight for each repetition. Typically, healthy participants show consistent kinematics when assessed at different time points [34,35].

### 2.4. Derivation of Kinematics

**Figure 2:**
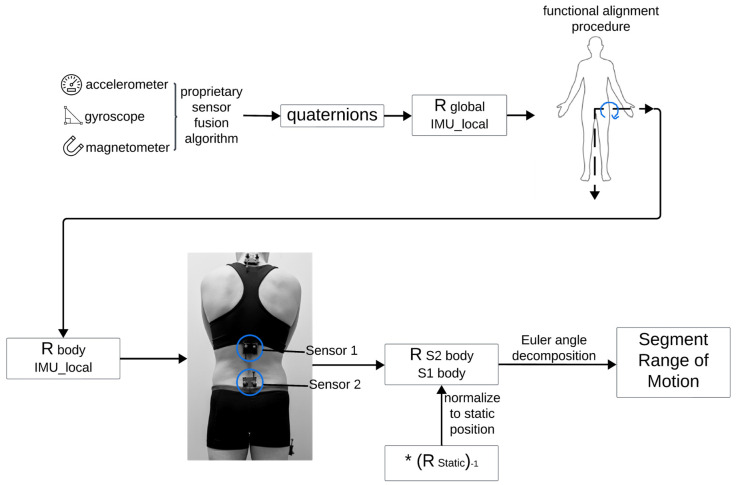
Flowchart outlining derivation of kinematics for IMU data. Figure was created using Lucidspark (Lucid Software Inc., 2024, accessed 26 September 2024) and includes icons from BioRender (https://www.biorender.com/, accessed 26 September 2024) and Microsoft PowerPoint, version 16.89.1. The * represents matrix multiplication.

**Figure 3:**
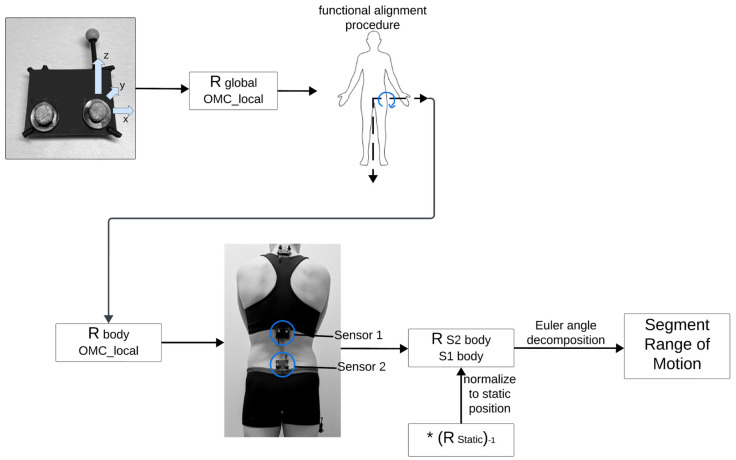
Flowchart outlining derivation of kinematics for OMC data. Figure was created using Lucidspark (Lucid Software Inc., 2024, accessed 26 September 2024) and includes icons from BioRender (https://www.biorender.com/, accessed 26 September 2024). The * represents matrix multiplication.

A custom algorithm (openly available from GitHub at https://github.com/anb254/Lifeware-IMU-Accuracy-Study, accessed 25 August 2024) was implemented in Matlab (Matlab R2023B, The MathWorks Inc., Natick, MA, USA) to quantify spine and hip kinematics obtained from both the IMU and OMC systems. Flowcharts for derivation of kinematics for OMC and IMU data are shown in Figure 2 and Figure 3. Kinematics were derived for three segments (lumbar, thoracic, and hip) across six movements, resulting in a total of 18 ‘segment movements’ for each participant. IMU quaternions were converted to 3 × 3 rotation matrices between the IMU sensor local coordinate system and the global coordinate system ($R_{\mathrm{IMU}\_\mathrm{local}}^{\mathrm{global}}$). Similarly, right-handed coordinate systems were derived from Vicon marker data using rigid body clusters placed on each IMU ($R_{\mathrm{OMC}\_\mathrm{local}}^{\mathrm{global}}$). To calculate ROM regarding functionally relevant axes, participants performed a modified functional alignment procedure [36,37] from which a rotation matrix was derived that would convert points from the local IMU or Vicon coordinate system to a ‘body’ coordinate system ($R_{\mathrm{IMU}\_\mathrm{local}}^{\mathrm{body}}\;\mathrm{or}\;R_{\mathrm{OMC}\_\mathrm{local}}^{\mathrm{body}}$). The alignment procedure was completed twice, once before Time 1 and once before Time 2. One repetition of trunk flexion defined the functional medial–lateral axis for the spine sensors and one repetition of hip flexion defined the functional medial–lateral axis for the hip sensor. The vertical axis for the ‘body’ coordinate system was defined for the IMUs using orientation with respect to gravity (pitch and roll) in a static pose and for Vicon data using the vertical vector from the Vicon-derived $R_{\mathrm{OMC}\_\mathrm{local}}^{\mathrm{global}}$ rotation matrix in a static pose. The third and final axis of the ‘body’ coordinate system (i.e., anterior–posterior axis) was derived by the cross product of the two previous axes (medial–lateral and vertical). A second cross product was taken to ensure orthogonality of the entire ‘body’ coordinate system.

Appropriate matrix multiplication was used to derive 3 × 3 rotation matrices representing the rotation from one sensor in its body coordinate system to a second sensor in its body coordinate system. Appropriate matrix multiplication is shown in Formula (1) (S# body = sensor body coordinate system; S# local = sensor local coordinate system). This same formula was used for both IMU and OMC rotation matrix calculations, with appropriate local coordinate systems (either IMU or OMC).

(1) $$R_{S1\;\mathrm{body}}^{S2\;\mathrm{body}}=R_{S2\;\mathrm{local}}^{S2\;\mathrm{body}}\cdot R_{\mathrm{global}}^{S2\;\mathrm{local}}\cdot R_{S1\;\mathrm{local}}^{\mathrm{global}}\cdot R_{S1\;\mathrm{body}}^{S1\;\mathrm{local}}$$

To account for individual differences in anatomy, final rotation matrices were normalized to each individual’s static position. Static position was defined as the last five frames of the recorded trial after completion of the movement task. To normalize rotation matrices to each individual’s static posture, final rotation matrices ($R_{S1\;\mathrm{body}}^{S2\;\mathrm{body}}$) were multiplied by the inverse of the rotation matrix derived from the static position. Euler angles were derived from this normalized $R_{S1\;\mathrm{body}}^{S2\;\mathrm{body}}$ rotation matrix according to the most appropriate order of decomposition. For AR, a transverse–sagittal–frontal (AR-F/E-SB) order was used. For SB, a frontal–sagittal–transverse (SB-F/E-AR) order was used. For FE, a sagittal–frontal–transverse (F/E-SB-AR) order was used. Resulting angles representing three-dimensional ROM between two sensors were derived.

### 2.5. Visual Inspection and Peak ROM Extraction

**Figure 4:**
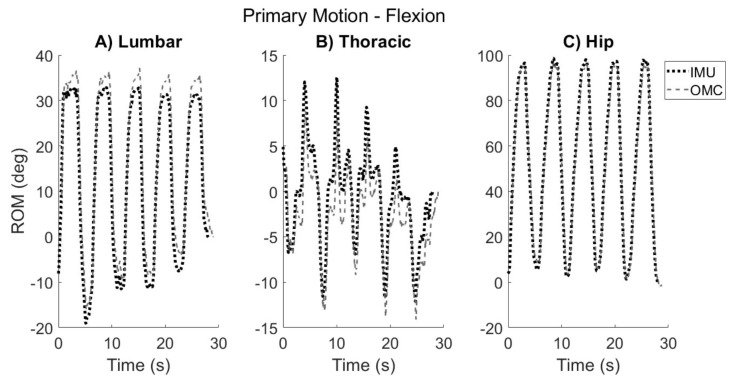
Typical kinematic trace of primary ROM that one participant demonstrated during flexion task in the sagittal plane for (**A**) lumbar, (**B**) thoracic, and (**C**) hip segments. The IMU kinematic trace is shown by the dotted black line, and the OMC trace is shown by the dashed gray line. Positive values correspond to forward flexion. Maximum ‘peak’ ROM was extracted for each segment for each of the five repetitions.

**Figure 5:**
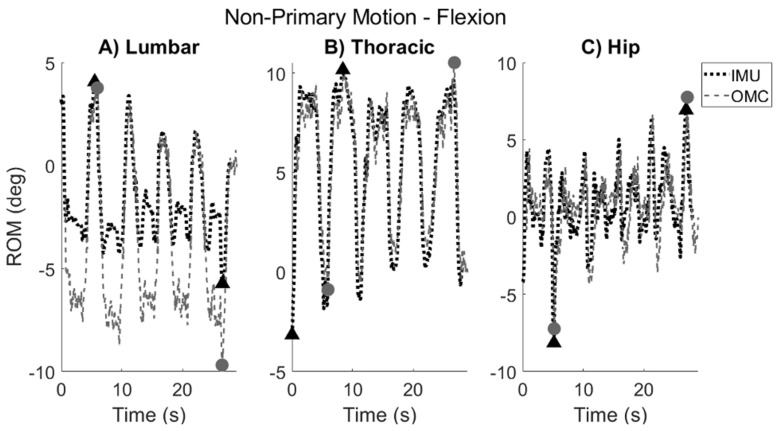
Typical kinematic trace of non-primary ROM that one participant demonstrated during flexion task in the the transverse plane for (**A**) lumbar, (**B**) thoracic, and (**C**) hip segments. The IMU kinematic trace is shown by the dotted black line, and the OMC trace is shown by the dashed gray line. Positive values correspond to left rotation. Maximum and minimum ROM achieved over entire trial (indicated by black triangles for IMU and gray circles for OMC) were extracted to derive non-primary ROM values.

Graphical representations of the ROM achieved during each segment movement were derived for all participants for both the IMU and OMC systems (Figure 4 and Figure 5). Each plot was visually inspected by the author (A.H.B.). The maximum ‘peak’ ROM achieved in the primary plane for each segment was extracted for each of the five repetitions of each movement. Distinct movement repetitions were visually identified by inspection of the kinematic trace of the T1 sensor in reference to the L5 sensor. In the case that other segments preceded or lagged behind the start of the T1/L5 kinematic trace, inspection of that particular segment was used to identify distinct repetitions for that segment. Secondary and tertiary authors (R.R. and K.M.B.) were consulted for consensus regarding any kinematic traces that did not clearly show visible differentiation between movement repetitions.

Average ‘peak’ ROM for each segment movement was calculated by averaging the peak ROM values achieved across all repetitions [11]. Six segment movements (out of 284, 2 percent) included only four repetitions. For these six segment movements, the fifth repetition was missing or excluded because either (1) the participant only performed four repetitions, or (2) there was a system error visualized in the kinematic trace.

Due to individual movement differences, some participants demonstrated spine segment movement in the direction opposite to the global direction of movement that was cued. For example, when performing left axial rotation of the trunk, most of the available motion is a result of the left rotation of the thoracic spine [38]. The lumbar spine may move in one of three ways depending on individual movement strategies and spine mobility. The lumbar segment may (1) rotate to the left (in the direction of cued movement), (2) remain in a neutral position as the participant tries to maintain a ’straight pelvis’ as instructed, or (3) rotate slightly to the right, again in an effort to maintain a straight pelvis. The third possibility, which will be termed ‘countermovement’, is not due to a misunderstanding of directions, but rather it is due to differences in individual movement strategies and spine mobility constraints. Whenever this ‘countermovement’ was observed in a kinematic trace, the authors confirmed the countermovement via video recording of the participant’s movement. Peak ROM for each repetition was then extracted for whichever direction the participant effectively moved their spine segment (rather than the direction they were cued to move).

ROM was also derived in the non-primary planes of movement (i.e., sagittal/frontal planes during AR, sagittal/transverse planes during SB, and frontal/transverse planes during F/E). Because coupled movement varies between participants [17], and it was not easy to predict its directionality, non-primary motion was not quantified for each repetition as was conducted for primary motion. Instead, non-primary motion was described as the maximum total ROM achieved over the total movement (i.e., |maximum positive ROM-minimum negative ROM|) in the secondary and tertiary planes (Figure 5).

### 2.6. Statistical Analysis

To determine repeated-measures reliability of the IMUs, ICC values were calculated to compare average peak ROM values between Trial 1 and Trial 2. Strength of agreement was based on the following ICC cutoffs: poor (<0.5), moderate (0.50–0.75), good (0.75–0.90), and excellent (>0.90) [39]. To determine accuracy of the IMUs as compared to the gold-standard OMC system, ICC and root-mean-squared-error (RMSE) values were calculated to compare average peak ROM values between the IMUs and OMC systems. Bland–Altman [40] analyses were performed to assess mean bias between the IMU and OMC systems for each segment movement, across all five repetitions. All analyses were performed in SPSS (version 28.0).

## 3. Results

### 3.1. Participants and Missing Data

Seventeen (17) participants consented to this IRB-approved study. The participant characteristics are described in Table 1. Sixteen (16) sets of participant data were analyzed for repeated-measures reliability and concurrent validity, with one participant excluded due to significant magnetometer fluctuations during the data collection. The reliability analysis included 15 participants; one participant was excluded due to unavailable IMU data at Time 2 due to battery depletion. One participant was excluded from hip segment F/E reliability analysis also due to battery depletion. One participant was excluded from lumbar and thoracic R AR validity analyses due to OMC marker occlusion of the T12/L1 sensor.

### 3.2. Repeated-Measures Reliability

In the primary plane, twelve segment movements showed good–excellent correlations (ICC = 0.77–0.96) and six segment movements showed moderate correlations based on the ICC values (Table 2). In the non-primary planes, eight segment movements in the secondary plane and six segment movements in the tertiary plane showed good–excellent correlations (Table 2). Ten segment movements in the secondary plane and twelve segment movements in the tertiary plane showed poor–moderate correlations.

### 3.3. Concurrent Validity

#### 3.3.1. Primary Motion

The majority of the correlations (ICC values) between the IMUs and OMC in the primary plane were good–excellent (ICC = 0.77–0.99), although four segment movements (out of eighteen) showed poor–moderate correlations (Table 3). The primary RMSE values between the two systems ranged from 3.03° to 15.75°. A full description of the ICCs and RMSEs for each segment movement in the primary plane is included in Table 3.

**Figure 6:**
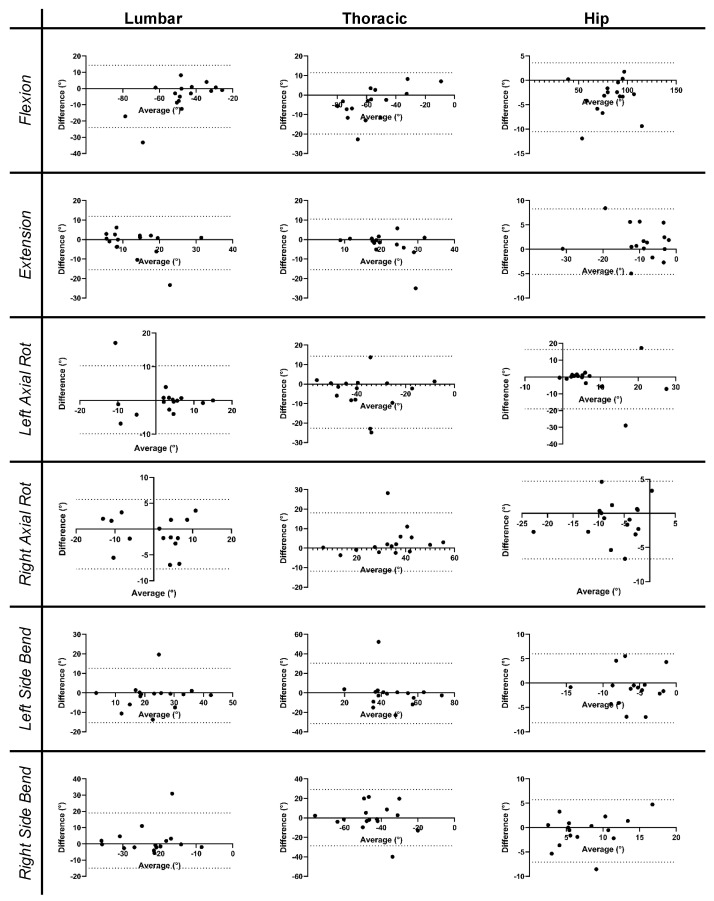
Bland–Altman plots corresponding to each ROM task (rows) for each segment (columns). Dotted lines on the individual plots correspond to two standard deviations above and below the line of equality (at y = 0). Rot = rotation.

The Bland–Altman plots [40] are shown in Figure 6, and the associated mean bias between the two systems is shown in Table 4. There was high variability in the system bias across the subjects, as indicated by the high standard deviation of the biases (Table 4). Some participants had biases that fell outside two standard deviations (Figure 6). The outliers identified from the Bland–Altman plots are listed in Table 5. Due to its squaring of errors, RMSE is heavily influenced by outliers. Therefore, a secondary analysis was performed in which the RMSE was recalculated after the outliers were excluded. The adjusted RMSE values are shown in Table 3. Under this secondary analysis, the primary RMSE values ranged from 2.44° to 10.30°.

**Table 3:** ICCs and RMSEs between IMU and OMC measurements (N = 16) during each movement for each spine segment in the primary plane of movement (i.e., flexion/extension = sagittal, side bend = frontal, axial rotation = transverse).

		ICC (CI, 95%)	RMSE (deg)	Adjusted RMSE (deg) ^#^
Flexion	Lumbar	0.88 (0.65–0.96) ^†^	10.65	6.69
	Thoracic	0.99 (0.96–1.00) ^†^	5.02	3.18
	Hip	0.99 (0.98–1.00) ^†^	4.89	3.88
Extension	Lumbar	0.77 (0.35–0.92) ^†^	7.00	3.87
	Thoracic	0.91 (0.75–0.97) ^†^	3.03	2.54
	Hip	0.95 (0.84–0.98) ^†^	3.67	3.00
Left Axial Rotation	Lumbar	0.88 (0.65–0.96) ^†^	5.00	2.60
	Thoracic	0.84 (0.55–0.94) ^†^	14.16	9.94
	Hip	0.66 (0.03–0.88)	8.82	2.63
Right Axial Rotation	Lumbar *	0.95 (0.86–0.98) ^†^	3.45	3.45
	Thoracic *	0.78 (0.33–0.93) ^†^	15.75	10.30
	Hip	0.93 (0.80–0.98) ^†^	2.98	2.98
Left Side Bend	Lumbar	0.87 (0.62–0.95) ^†^	7.02	5.01
	Thoracic	0.32 (−0.96–0.76)	9.70	3.81
	Hip	0.69 (0.12–0.89)	3.66	3.66
Right Side Bend	Lumbar	0.67 (0.06–0.89)	8.62	3.82
	Thoracic	0.80 (0.42–0.93) ^†^	7.28	3.10
	Hip	0.84 (0.53–0.94) ^†^	3.24	2.44

deg = degrees; * N = 15; ^†^ indicates good–excellent ICCs; ^#^ RMSE was recalculated after outliers (listed in Table 5) were excluded.

**Table 4:** Bias between IMU and OMC systems in the primary plane as determined by Bland–Altman analysis (N = 16).

	Lumbar	Thoracic	Hip
Flexion	−4.9 (9.8)	−0.5 (5.2)	−3.4 (3.6) *
Extension	−1.8 (7.0)	−0.6 (3.1)	1.6 (3.4)
Left Axial Rotation	0.2 (5.2)	7.4 (12.5)	−1.3 (9.0)
Right Axial Rotation	−1.0 (3.4) *	−7.2 (13.4) *	−1.0 (2.9)
Left Side Bend	−1.3 (7.1)	2.2 (9.8)	−1.0 (3.6)
Right Side Bend	2.0 (8.7)	−2.7 (7.0)	−0.7 (3.3)

Values were derived as mean peak IMU ROM – mean peak OMC ROM and are listed as mean (standard deviation), * N = 15.

**Table 5:** Outliers from Bland–Altman analysis for primary movement.

Movement	Participant (Segment(s))
Flexion	S02 (lumbar)
	S04 (thoracic)
	S16 (hip)
Extension	S02 (lumbar)
	S05 (thoracic)
	S12 (hip)
Left Axial Rotation	S05 (thoracic)
	S12 (lumbar, hip)
	S16 (hip)
Right Axial Rotation	S05 (thoracic)
Left Side Bend	S05 (lumbar, thoracic)
Right Side Bend	S02 (lumbar)
	S05 (thoracic)
	S06 (hip)
	S18 (thoracic)

Outliers are defined as biases that are more than two standard deviations from the mean.

#### 3.3.2. Non-Primary Motion

The ICC and RMSE values comparing the two systems in the non-primary planes are shown in Table 6. In the secondary plane, seven segment movements (out of eighteen) showed good–excellent correlations between the systems, while the others had poor–moderate correlations. In the tertiary plane, six segment movements had good–excellent correlations between the systems, while the other segment movements had poor–moderate correlations.

The RMSE values between the IMU and OMC systems ranged from 2.19° to 27.30° in the secondary plane and 3.21° to 45.88° in the tertiary plane. The lumbar-F segment movement was associated with particularly large RMSE values. Upon further inspection, it was discovered that these large RMSE values were heavily influenced by just one participant, for whom the IMU showed non-physiological ROM values (i.e., 85.7° primary, 79.8° secondary, and 131.9° tertiary). Upon removing this participant’s lumbar-F data, the RMSE for lumbar-F was reduced from 27.30° and 45.88° to 21.86° and 28.14° in the secondary and tertiary planes, respectively. The Bland–Altman plots were not created to compare the system bias for the non-primary movements given the poor–moderate correlation and higher error between the two systems in the non-primary planes.

## 4. Discussion

The purpose of this study was to evaluate the repeated-measures reliability and concurrent validity of an inertial measurement unit system to measure the three-dimensional spine/hip kinematics for six ROM tasks. This study assessed the thoracic, lumbar, and hip segments during six different movements, resulting in a total of 18 segment movements for each participant. The IMUs had good–excellent reliability for the majority of the segment movements in the primary plane and poor–moderate reliability for the majority of the segment movements in the non-primary planes. The correlation between the IMU and OMC systems was mostly good–excellent in the primary plane and poor–moderate in the non-primary planes. The majority of the primary-plane RMSE values fell below a 10-degree threshold, which is comparable to other IMU validity studies [12,41,42,43]. Larger RMSE values were found for several segment movements in the non-primary planes. The Bland–Altman analysis showed reasonable agreement between the two systems although high variability among the participants, with several participants falling outside two standard deviations of the mean bias in the system. The removal of these ‘outlier’ participants resulted in a reduction in the RMSE values. The presence of outliers points toward the use of these sensors for population-based studies rather than individual clinical assessment.

### 4.1. Repeated-Measures Reliability

A primary aim of this study was to determine the repeated-measures reliability of the IMUs. We assumed that our repeated-measures reliability analysis would be indicative of the sensor performance rather than the differences in participant movement between Time 1 and Time 2 because healthy participants typically have shown consistent primary-plane movement when assessed at different time points [34,35]. Overall, the IMUs showed good–excellent reliability in the primary plane for 12/18 segment movements. The remaining six segment movements showed moderate reliability. The moderate agreement for the extension movement was not surprising based on the protocol adaptations to minimize OMC marker artifact. Specifically, when the participants completed maximum extension, the L1 and L5 sensor markers often obscured each other, so the participants were cued to only extend partway through the full range to prevent artifact. This may have induced altered motions that were not easily replicated between Time 1 and Time 2, thereby contributing to the lower ICCs for primary plane extension. The moderate agreement for the other segment movements (i.e., hip-L AR, thoracic-L SB, hip-L SB, and lumbar-R SB) cannot be explained by the movement variability introduced through protocol modifications. Due to the rigorous protocol ensuring consistent movement between the trials (i.e., the participants were provided the opportunity to practice the movements before data collection and were instructed to move as far as possible for each movement), we assume that the moderate agreement for these segment movements is indicative of the true sensor performance. However, because we did not derive the gold-standard ROM values at Time 1 and Time 2, we cannot rule out the possibility that the ICC differences may be due to true movement differences between the trials.

Despite the good performance in the primary plane, the IMUs showed poor–moderate reliability in the non-primary planes. Previous studies indicate that individuals have less consistent movements in non-primary planes [34,35], suggesting that the lower ICC values in the non-primary planes for the current study may be partially due to actual movement differences between Time 1 and Time 2. Another explanation for the low ICC values in the non-primary planes may be due to the smaller overall movement in these planes. The smaller movement may have contributed to the low inter-subject variability in the non-primary planes, which is known to produce low ICCs, even in data sets that are similar [34,44]. Overall, it is unclear whether the low ICCs for the non-primary planes in the current study were due to poor reliability of the sensors themselves or other contributing factors. Future studies may wish to assess sensor reliability in parallel with a gold-standard system or use a controlled passive movement device to systematically evaluate non-primary-plane movement.

### 4.2. Concurrent Validity

Another primary aim of this study was to determine the concurrent validity of the IMUs as compared to a gold-standard OMC system. The ICC values suggest that the correlation between the IMUs and OMC system was generally good–excellent in the primary plane and poor–moderate in the non-primary planes. The results are consistent with previous literature showing lower system agreement for non-primary planes [17,18,45,46]. The smaller magnitude of movement that occurs in non-primary planes likely contributes to a depressed signal-to-noise ratio and thereby inferior performance of the IMUs in quantifying non-primary plane motion [18,45,46]. It is important to note that many ICC values were associated with large confidence intervals, pointing toward significant variability in system agreement between the participants. Previous studies have also noted significant variability in system agreement between participants [47], which may be due to inconsistent calibration, anomalies in the built-in sensor fusion algorithm [45], or skin/clothing artifact [13].

A comparison of the RMSE values in the primary and non-primary planes supports the conclusions drawn from the corresponding ICC values: the IMUs show acceptable validity in the primary plane, but the results are less conclusive for the non-primary planes. Typically, RMSE values up to 5° are considered to be clinically acceptable [12]. A systematic review found the average RMSE for the IMU vs gold standard comparison to be <2.4° for the lumbar spine in the primary plane [11]. However, in the current study, many of the segment movements fell outside the 5° range for primary-plane movement, pointing toward limited usability of these IMUs for clinical interpretation [12]. Despite the recommended 5° cutoff for clinical interpretation, realistically, many studies report RMSE values up to 10° when comparing the measurement errors of IMUs to OMC [12,41,42,43,48]. For instance, one study reported RMSE values up to 40° depending on the joint and plane of movement [43]. In the current study, only one segment movement in the primary plane was associated with an RMSE > 10° after the exclusion of outliers (Table 3, ‘Adjusted RMSE’ column). Therefore, based on the 10° threshold [12,41,42,43,48], the IMUs in the current study may be acceptable substitutes for OMC when measuring the ROM in the primary plane, but their acceptability for non-primary plane ROM is not yet well-supported across multiple segment movements.

The IMUs used in the current study may be best suited for aggregate research or population-based studies rather than for clinical interpretation or individual assessment, which tend to require a higher degree of accuracy [12]. Population-based studies rely on group averages and are more robust to outliers due to larger sample sizes. We hypothesize that the outlier values may have been the result of inter-individual differences in the accuracy of the functional calibration procedure, anomalies in the IMU’s built-in sensor fusion algorithm [45], or clothing/skin artifact [13]. This hypothesis is based on the following observations: (1) a small number of participants contributed to the majority of the outliers and (2) the outlier values were associated with intact raw data, suggesting inconsistencies in sensor fusion or other down-stream processing. However, due to the proprietary nature of the built-in sensor fusion algorithm, additional information regarding the sensor fusion could not be obtained, which is a limitation of using a built-in sensor fusion algorithm. To ensure rigorous accuracy testing, future researchers may consider developing their own sensor fusion algorithm, although this must be balanced with the extensive time and expertise such a task would require, thereby reducing the feasibility of use across a variety of settings. Although still available for purchase and commonly used for joint angle measurement [49,50,51], the BNO055 has recently been discontinued and newer BHIxy products may be used as replacements. Future research should investigate the accuracy of updated models and identify specific causes for outlier values.

To the authors’ best knowledge, there are no comparable studies that systematically assess the reliability and validity of IMUs to measure three-dimensional multi-segmental spine angles across clinical ROM tasks. This innovative study is the first that uses IMUs to thoroughly capture the three-dimensional kinematics of the spine and hip segments. Three-dimensional joint angles provide insight into compensatory patterns and coupled movement strategies, which may perpetuate pain and disability [24,25]. Additionally, the importance of multi-segmental assessment has been emphasized in the literature [7,9,19]; thus, a multi-sensor system such as the one described here should be the standard moving forward. The presence of movement differences associated with LBP is not sufficient to inform clinical practice. Instead, the future work must explore the clinical correlates to the observed movement impairments [10]. IMUs such as those used in the current study enable the remote monitoring of spinal motion and posture, which facilitates increasingly accurate depiction of the associations between movement impairments, symptomology, and daily function.

### 4.3. Limitations

The current study investigated the reliability and accuracy of IMU sensors in a back-healthy population; the results may be different in individuals with low back pain or with different adiposities. Future work should examine the reliability and accuracy of these sensors for use in clinical populations. The study protocol described here is also associated with some minor limitations. For instance, the IMU and OMC systems were not synchronized. However, peak extraction does not require time synchronization, and the current study did not assess temporal accuracy. Instead, the extraction of the peak ROM values relied on the visual inspection of the kinematic traces by the authors, which presents its own limitations and is inherently prone to human error. To reduce human error and improve consistency, automated processes were used to extract the peak ROM, and the visual inspection was performed by one author (A.H.B.) who consulted with secondary and tertiary authors (R.R. and K.M.B.) as needed. Additionally, IMUs are prone to position drift over time, which may have made the peak extraction less reliable, especially over multiple movement repetitions. The authors of the current study opted to extract the peak ROM values for improved clinical relevance and to be consistent with the previous literature [11]. The agreement between the IMU and OMC systems may be improved by correcting for the drift after each movement repetition or examining the total movement excursion (i.e., L AR ROM + R AR ROM) rather than the peak ROM. The positioning of the OMC markers on the sensor itself presents a final limitation. The short distance between the markers and the protruding arm of the plastic case (Figure 1) to which the third marker was adhered introduce the potential for additional marker/skin artifact, a portion of which is unavoidable within OMC systems [13,14].

## 5. Conclusions

The current study investigated the reliability and accuracy of IMUs to determine the three-dimensional angles of the thoracic, lumbar, and hip segments during six different clinical ROM tests. This study is the first of its kind to assess the three-dimensional ROM across multiple spine/hip segments for a thorough assessment of the kinematics. The results of the current study suggest that these IMUs may be reasonably used to replace OMC for measuring ROM in the primary plane of movement. The presence of outliers in the validity analysis suggests that these sensors are best suited for population-based studies, which are more robust to outliers, rather than for individual clinical use. To identify the potential reasons for outlier values, future work should investigate inter-participant differences in the calibration, accuracy of the built-in sensor-fusion algorithm, and skin/clothing artifact. Given their utility for population-based studies, these IMUs are particularly relevant for large phenotyping studies, many of which seek to combine rich biomechanical data sets from large populations with biopsychosocial parameters and multi-omics [10,52]. Large phenotyping studies hold promise for the future of precision medicine as applied to LBP [10,20]. Insights from the current study may inform the interpretation of the kinematic data collected as part of such large phenotyping studies.

## Figures and Tables

**Table 1:** Participant descriptors for participants enrolled in study (N = 17).

Age	32.3 (14.7) years
Sex	11 Female, 6 Male
BMI	25.7 (7.5)
Race	70.6 (12) White/17.6 (3) Asian/11.8 (2) Black

Age and BMI are listed as mean (standard deviation). Race is listed as percent (number).

**Table 2:** ICCs comparing IMUs at two different time points on same day (N = 15).

		Primary	Secondary	Tertiary
Flexion	Lumbar	0.77 (0.30–0.92) ^†^	0.02 (−1.9–0.67)	0.10 (−1.67–0.70)
	Thoracic	0.96 (0.88–0.99) ^†^	0.44 (−0.66–0.81)	0.63 (−0.12–0.87)
	Hip *	0.92 (0.75–0.97) ^†^	0.66 (−0.07–0.89)	0.77 (0.28–0.93) ^†^
Extension	Lumbar	0.66 (−0.03–0.88)	−0.39 (−3.15–0.53)	0.60 (−0.20–0.87)
	Thoracic	0.86 (0.58–0.95) ^†^	0.79 (0.38–0.93) ^†^	0.61 (−0.16–0.87)
	Hip *	0.73 (0.16–0.91)	0.08 (−1.87–0.71)	0.78 (0.32–0.93) ^†^
Left Axial Rot	Lumbar	0.86 (0.57–0.95) ^†^	0.80 (0.39–0.93) ^†^	0.75 (0.25–0.92) ^†^
	Thoracic	0.94 (0.82–0.98) ^†^	0.35 (−0.94–0.78)	0.94 (0.82–0.98) ^†^
	Hip	0.59 (−0.21–0.86)	0.98 (0.93–0.99) ^†^	0.86 (0.57–0.95) ^†^
Right Axial Rot	Lumbar	0.91 (0.73–0.97) ^†^	0.49 (−0.51–0.83)	0.17 (−1.47–0.72)
	Thoracic	0.94 (0.81–0.98) ^†^	0.80 (0.39–0.93) ^†^	0.55 (−0.35–0.85)
	Hip	0.83 (0.51–0.94) ^†^	0.79 (0.39–0.93) ^†^	0.59 (−0.22–0.86)
Left Side Bend	Lumbar	0.93 (0.80–0.98) ^†^	0.62 (−0.19–0.88)	−0.23 (−2.83–0.61)
	Thoracic	0.73 (0.20–0.91)	0.87 (0.59–0.96) ^†^	0.61 (−0.23–0.87)
	Hip	0.58 (−0.26–0.86)	0.57 (−0.34–0.86)	0.75 (0.23–0.92) ^†^
Right Side Bend	Lumbar	0.73 (0.19-0.91)	0.31 (−1.07–0.77)	0.11 (−1.67–0.70)
	Thoracic	0.82 (0.47–0.94) ^†^	0.84 (0.53–0.95) ^†^	0.73 (0.19–0.91)
	Hip	0.83 (0.50–0.94) ^†^	0.83 (0.50–0.94) ^†^	0.16 (−1.51–0.72)

Values are listed as ICC (95% confidence interval). Primary/secondary/tertiary movements are as follows: flexion/extension = sagittal/frontal/transverse; side bend = frontal/sagittal/transverse; axial rotation = transverse/sagittal/frontal. Rot=rotation. * N = 14. ^†^ indicates good–excellent ICC values.

**Table 6:** ICC and RMSE values between IMU and OMC measurements (N = 16) during each movement for each spine segment in the non-primary planes of movement (i.e., secondary: flexion/extension = frontal, side bend = sagittal, axial rotation = sagittal; tertiary: flexion/extension = transverse, side bend = transverse, axial rotation = frontal).

		Secondary Plane		Tertiary Plane	
		ICC (CI, 95%)	RMSE (deg)	ICC (CI, 95%)	RMSE (deg)
Flexion	Lumbar	0.20 (−1.28–0.72)	27.30 ^#^	−0.07 (−2.06–0.63)	45.88 ^##^
	Thoracic	0.74 (0.25–0.91)	6.06	0.38 (−0.78–0.78)	10.61
	Hip	0.68 (0.07–0.89)	6.41	0.85 (0.58–0.95) ^†^	4.24
Extension	Lumbar	0.23 (−1.19–0.73)	13.19	0.75 (0.29–0.91) ^†^	3.46
	Thoracic	0.43 (−0.64–0.80)	3.86	0.58 (−0.20–0.85)	4.21
	Hip	0.78 (0.36–0.92) ^†^	2.81	0.08 (−1.64–0.68)	11.29
Left Axial Rotation	Lumbar	0.93 (0.81–0.98) ^†^	2.19	0.76 (0.32–0.92) ^†^	3.57
	Thoracic	0.54 (−0.33–0.84)	9.79	0.23 (−1.20–0.73)	18.84
	Hip	0.59 (−0.16–0.86)	12.88	0.26 (−1.12–0.74)	11.99
Right Axial Rotation	Lumbar *	0.69 (0.06–0.89)	5.28	0.43 (−0.69–0.81)	8.72
	Thoracic *	0.95 (0.84–0.98) ^†^	3.40	0.12 (−1.61–0.71)	22.68
	Hip	0.93 (0.80–0.98) ^†^	4.62	0.42 (−0.66–0.80)	4.28
Left Side Bend	Lumbar	0.53 (−0.39–0.84)	9.51	0.50 (−0.48–0.83)	7.34
	Thoracic	0.85 (0.57–0.95) ^†^	3.96	0.85 (0.55– 0.95) ^†^	7.73
	Hip	0.92 (0.76–0.97) ^†^	3.11	0.92 (0.76–0.97) ^†^	3.21
Right Side Bend	Lumbar	0.54 (−0.32–0.84)	7.94	0.56 (−0.25–0.85)	6.39
	Thoracic	0.74 (0.25–0.91)	6.56	0.89 (0.69–0.96) ^†^	5.52
	Hip	0.91 (0.74–0.97) ^†^	2.95	0.08 (−1.65–0.68)	6.05

ICCs are listed with 95% confidence intervals; deg = degrees; * N = 15; ^†^ good–excellent ICCs; ^#^ RMSE reduced to 21.86° when single subject’s non-physiological data were excluded; ^##^ RMSE reduced to 28.14° when single subject’s non-physiological data were excluded.

## Data Availability

The custom code presented in the study is openly available from GitHub at https://github.com/anb254/Lifeware-IMU-Accuracy-Study, accessed 25 August 2024. The raw data supporting the conclusions of this article will be made available by the authors upon request.
